# Click-chemistry-based protocol for detecting 4-octyl-itaconate-alkylated proteins in primary mouse macrophages

**DOI:** 10.1016/j.xpro.2024.103314

**Published:** 2024-09-19

**Authors:** Chaofei Su, Tian Cheng, Hanyi Zhang, Hang Yin

**Affiliations:** 1 State Key laboratory of Membrane Biology, School of Pharmaceutical Sciences, Tsinghua-Peking Center for Life Sciences, Key Laboratory of Bioorganic Phosphorous Chemistry and Chemical Biology (Ministry of Education), Tsinghua University, Beijing 100084, China

**Keywords:** Cell-based Assays, Immunology, Molecular/Chemical Probes, Chemistry

## Abstract

4-Octyl itaconate (4-OI), a derivative of itaconate, inhibits inflammation by alkylating its target proteins. Here, we present a click-chemistry-based protocol for detecting 4-OI-alkylated proteins in mouse primary bone-marrow-derived macrophages (BMDMs) by using an itaconate-alkyne (ITalk) probe. We describe steps for culturing and treating BMDMs and details on using click chemistry in the cell lysate. We also detail procedures for detecting alkylated proteins by western blot.

For complete details on the use and execution of this protocol, please refer to Su et al.^1^

## Highlights

• Steps for isolating and culturing primary bone-marrow-derived macrophages
• Instruction for click chemistry in primary BMDMs
• Procedure to detect 4-octyl-itaconate-alkylated proteins by immunoblotting

## Abstract (editor highlights)

Publisher’s note: Undertaking any experimental protocol requires adherence to local institutional guidelines for laboratory safety and ethics.

## Graphical abstract

**Figure:**
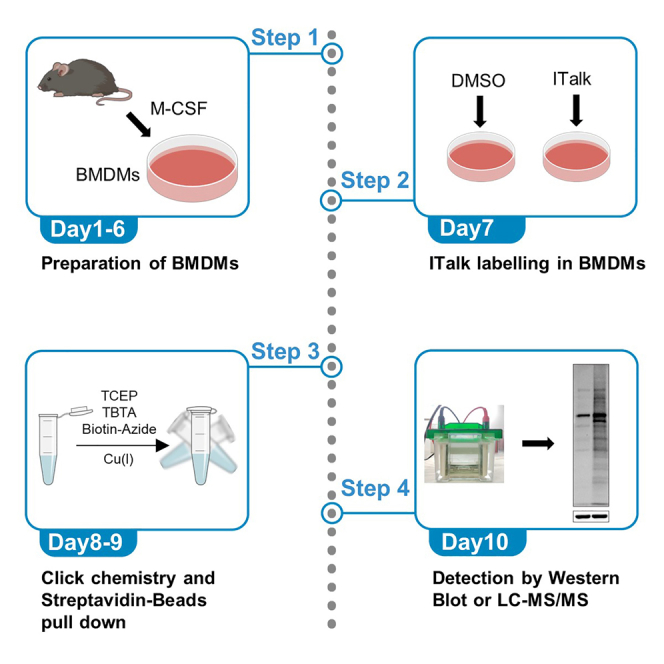

## Before you begin

Itaconate, an electrophilic α, β-unsaturated carboxylic acid has been reported to inhibit the inflammation responses in many cells.^2^ It can alkylate protein cysteine residues by Michael addition to regulate the function of targeted proteins.^3^ 4-Octyl itaconate (4-OI), a derivative of itaconate, also inhibits the inflammation by alkylating its targeted proteins.^3^ Itaconate alkyne (ITalk) is a reported biorthogonal probe to detect itaconate alkylation in living cells by click chemistry.^4^ Click chemistry was proposed as a functional form of chemistry, where molecules snap together quickly and efficiently in a mild condition.^5^ The one of the most important reactions of click chemistry is the copper catalyzed azide-alkyne cycloaddition (CuAAC).^6^^,^^7^ This reaction is widely used and improved to link molecules together in many biorthogonal methods. The key rational of the ITalk is that the long carbon chain of 4-OI remains on the target proteins after modification.^4^ Therefore, the long carbon chain of 4-OI can provide the site for introducing the biorthogonal handle, such as alkyne.^4^ Consequently, we propose that ITalk should be referred to as a 4-OI alkylation probe instead of an itaconate alkylation probe. Here, we describe a click-chemistry-based protocol for detecting 4-octyl-itaconate-alkylated proteins in primary bone marrow-derived macrophages (BMDMs) via probe ITalk. The protocol below describes the specific steps for using BMDMs. However, we have also used this protocol in human THP-1, mouse Raw264.7 cells and mouse embryonic fibroblasts. What’s more, we have also used this protocol for detecting protein palmitoylation or succination in BMDMs via other metabolic labeling probes such as 17-ODYA^8^ or MMF-Yne.^9^

### Institutional permissions

All animal experiments were approved by the Institutional Animal Care Use Committees of Tsinghua University. Mice were kept in specific pathogen free (SPF) environment in Laboratory Animal Resources Center, Tsinghua University. Here we also remind our readers that if they want to get BMDMs, they will need to acquire permissions from the relevant institutions.

### Preparation of BMDMs

**Timing: 6 days**

1. Euthanize a C57BL/6J mouse using the approved methods of primary and secondary euthanasia at your institution.

***Note:*** The most widely used methods include the use of carbon dioxide, isoflurane overdose, or avertin overdose. The primary method should be followed by a secondary physical method, such as cervical dislocation.

2. Spray the entire mouse with 75% ethanol.

***Note:*** The dissection should be performed in a laminar flow hood.

3. Dissect out femur and tibia bones, remove major muscles around the bones and place bones in ice cold PBS as described previously by Toda and colleagues.^10^^,^^11^
4. In a biosafety cabinet, soak the bones in 75% ethanol for 1 min, then transfer bones in new sterile PBS in a 60 mm tissue culture dish, cut off extremities and flush them with ice cold PBS using a syringe and a 25G needle as described previously.^10^^,^^11^
5. Collect bone marrow in a 50 mL conical tube. Centrifuge at 500 × *g*, 4 min to pellet the bone marrow and discard the PBS.
6. Add 5 mL pre-warmed DMEM culture medium to the bone marrow pellets and homogenize the cell suspension by pipetting up and down.
7. Pass cell suspension through a 70-μm cell strainer on top of a new sterile 50 mL conical tube.
8. Plate the cell suspension to a Thermo Scientific Nunc EasYDish 150 mm dish. Typically, we can isolate about 20 million bone marrow cells from one mouse and seed bone marrow cells from one mouse to one 150 mm dish with total 25 mL DMEM culture medium.
9. Add mouse M-CSF into the culture medium at a final concentration of 40 ng/mL. This time is set to day 0. At day 2, add mouse M-CSF into the culture medium at a final concentration of 20 ng/mL.
10. Culture bone marrow cells in a standard humidified 37°C, 5% CO2 incubator. And collect cells on day 5.

***Note:*** Alternative protocols have been described for the generation of BMDMs, such as using the L929-conditioned medium.^10^ We have tested the alternative protocols and the test results are the same.

## Key resources table

**Table:** 

REAGENT or RESOURCE	SOURCE	IDENTIFIER
**Antibodies**		

Mouse anti-β-Actin (8H10D10) (1:3,000)	Cell Signaling Technology	Cat#3700, RRID: AB_2242334
Goat anti-mouse IgG-HRP (1:3,000)	Bio-Rad	Cat#1706516
Rabbit anti-STING/TMEM173 (1:1,500)	Proteintech	Cat#19851-1-AP, RRID: AB_10665370
Goat anti-rabbit IgG-HRP (1:3,000)	Bio-Rad	Cat#1706515
SA-HRP (1:1,500)	Beyotime Biotechnology	Cat#A0303

**Chemicals, peptides, and recombinant proteins**		

Itaconate-alkyne (ITalk)	MedChemExpress	Cat#HY-133870
Protease inhibitor cocktail (EDTA free, 100× DMSO)	Selleck	Cat#B14001
Streptavidin agarose resin	Thermo Fisher Scientific	Cat#20347
PBS	Thermo Fisher Scientific	Cat#C10010500
DMEM	Thermo Fisher Scientific	Cat#C11995500
FBS	Thermo Fisher Scientific	Cat#10099141
Penicillin-Streptomycin (100×)	TransGen Biotech	Cat#FG101-01
SDS	Solarbio Life Sciences	Cat#S8010
M-CSF	Novoprotein	Cat#CB34
Tris	Solarbio Life Sciences	Cat#T8060
6× Protein loading	TransGen Biotech	Cat#DL101-02
DMSO	Sigma-Aldrich	Cat#D8418
TBTA ligand	Sigma-Aldrich	Cat#678937
TCEP	Beyotime Biotechnology	Cat#ST045
Biotin-Azide	Sigma-Aldrich	Cat#762024
Cu_2_SO_4_	Sigma-Aldrich	Cat#451657
30% (w/v) Acrylamide:bisacrylamide (29:1) solution	Solarbio Life Sciences	Cat#A1010
1.5 M Tris-HCl buffer (pH 8.8)	Solarbio Life Sciences	Cat#T1010
1 M Tris-HCl buffer (pH 6.8)	Solarbio Life Sciences	Cat#T1020
Ammonium persulfate (APS)	Sigma-Aldrich	Cat#A3678
N,N,N,N′-tetramethyl-ethylenediamine (TEMED)	Beyotime Biotechnology	Cat#ST728
20× TBS buffer	Solarbio Life Sciences	Cat#T1080
Tween 20	Beijing BioDee Biotechnology	Cat#0777
Skim milk	Solarbio Life Sciences	Cat#D8340
Glycine	Solarbio Life Sciences	Cat#G8200
PageRuler prestained protein ladder	Thermo Fisher Scientific	Cat#26617

**Critical commercial assays**		

SuperSignal West Pico PLUS Substrate Kit	Thermo Fisher Scientific	Cat#34578
Pierce BCA Protein Assay Kit	Thermo Fisher Scientific	Cat#23225

**Experimental models: Organisms/strains**		

*Mus musculus*: C57BL/6J, adult (7–12 weeks), male	Tsinghua University	N/A

**Other**		

Thermo Scientific Nunc EasYDish 150 mm dish	Thermo Fisher Scientific	Cat#150462
Thermo Scientific Nunc EasYDish 60 mm dish	Thermo Fisher Scientific	Cat#150468
25G sterile hypodermic needles	Becton Dickinson	Cat#300600
70 mm cell strainers	Becton Dickinson	Cat#352350
Bioruptor plus	Diagenode	Cat#Bioruptor UCD-300 TO
iBright 1500	Thermo Fisher Scientific	Cat#A44115
Eppendorf ThermoMixer C	Eppendorf	Cat#5382000074

## Materials and equipment

**Table:** IP lysis buffer

Reagent	Final concentration	Amount
Tris	50 mM	6.04 g
NaCl	150 mM	8.76 g
Glycerol	10% (v/v)	100 mL
TritonX-100	0.2% (v/v)	2 mL
ddH_2_O	N/A	To 1000 mL
**Total**	**N/A**	**1000 mL**

Use HCl to adjust pH to 7.5, store at 4°C for up to 6 months. Sterilization is not required.

**Note:** All the ddH_2_O used in this protocol is the distilled deionized water that has a resistivity up to 18.2 MΩ cm.

**Table:** DMEM culture medium

Reagent	Final concentration	Amount
DMEM	N/A	445 mL
Heat-inactivated FBS	10%	50 mL
Penicillin-Streptomycin	1×	5 mL
**Total**	**N/A**	**500 mL**

FBS is heat-inactivated at 56°C for 20 min, filter-sterilized (0.22 μm) and stored at −20°C. DMEM culture medium is stored at 4°C for up to 2 months.

**Table:** 1× Protein Loading buffer

Reagent	Final concentration	Amount
IP lysis buffer	N/A	494 μL
6× Protein loading	1×	100 μL
100× protease inhibitors	1×	6 μL
**Total**	**N/A**	**600** μ**L**

Storage at 4°C and use within one day of preparation.

**Table:** 1.2% SDS/PBS

Reagent	Final concentration	Amount
SDS	1.2%	1.2 g
PBS	N/A	To 100 mL
**Total**	**N/A**	**100 mL**

Storage at 18°C–27°C for up to 6 months.

**Table:** 0.2% SDS/PBS

Reagent	Final concentration	Amount
SDS	0.2%	0.2 g
PBS	N/A	To 100 mL
**Total**	**N/A**	**100 mL**

Storage at 18°C–27°C for up to 6 months.

**Table:** 10% SDS

Reagent	Final concentration	Amount
SDS	10%	10 g
ddH_2_O	N/A	To 100 mL
**Total**	**N/A**	**100 mL**

Storage at 18°C–27°C for up to 6 months.

**Table:** 10% APS

Reagent	Final concentration	Amount
APS	10%	1 g
ddH_2_O	N/A	To 10 mL
**Total**	**N/A**	**10 mL**

Storage at 4°C for up to 1 month.

**Table:** 10× running buffer

Reagent	Final concentration	Amount
Tris	0.25 M	30.2 g
Glycine	1.92 M	144.1 g
SDS	1.0% (w/v)	10 g
ddH_2_O	N/A	To 1000 mL
**Total**	**N/A**	**1000 mL**

Store at 18°C–27°C for up to 6 months. During SDS-PAGE, dilute the 10× running buffer to 1× running buffer with ddH_2_O.

**Table:** 10× transfer buffer

Reagent	Final concentration	Amount
Tris	0.25 M	30.2 g
Glycine	1.92 M	144.1 g
ddH_2_O	N/A	To 1000 mL
**Total**	**N/A**	**1000 mL**

Store at 18°C–27°C for up to 6 months.

**Table:** 1× transfer buffer

Reagent	Final concentration	Amount
10× transfer buffer	1×	100 mL
Methanol	N/A	200 mL
ddH_2_O	N/A	To 1000 mL
**Total**	**N/A**	**1000 mL**

When do transfer, dilute the 10× transfer to 1× transfer with ddH_2_O and methanol and use the 1× transfer immediately.

**Table:** TBST buffer

Reagent	Final concentration	Amount
20× TBS buffer	1×	50 mL
Tween-20	0.1% (v/v)	1 mL
ddH_2_O	N/A	To 1000 mL
**Total**	**N/A**	**1000 mL**

Store at 18°C–27°C for up to 6 months.

**Table:** Blocking buffer

Reagent	Final concentration	Amount
Skim Milk	5% (w/v)	2.5 g
TBST buffer	N/A	To 50 mL
**Total**	**N/A**	**50 mL**

Storage at 4°C for up to 1 day.

## Step-by-step method details

### ITalk labeling in BMDMs

**Timing: 2 days**

This step describes the treatment of BMDMs with TIalk.

1. Pre warm the DMEM culture medium in a 37°C water bath.
2. After 5 days of differentiation, harvest and plate cells into Thermo Scientific Nunc EasYDish 60 mm dishes.
  a. Discard the culture medium of BMDMs.
  b. Add 5 mL PBS gently along the dish wall; shake the dish 5–10 times gently then discard the PBS. Repeat the step for one time to totally remove the red blood cells and suspension cells.
  c. Add 5 mL DMEM culture medium into the dish, then use cell scraper to scrap the BMDMs.
  d. Collect the BMDMs into a new 50 mL tube, then wash the dish with another 5 mL medium. Collect the medium into the tube, too.
  e. Count the BMDMs using blood cell counting chamber and dilute the BMDMs with culture medium to a final concentration of 1 × 10^6^ cells/mL.
  f. Prepare two 60 mm tissue culture dishes. Add 5 mL BMDMs suspensions into each 60 mm tissue culture dish. Add mouse M-CSF into the medium at a final concentration of 10 ng/mL.
3. Culture the cells in a standard humidified 37°C, 5% CO2 incubator for about 8 h to let the cell to adhere.

**CRITICAL:** It is necessary to warm the medium to decrease the effects of changing medium.

4. Prepare two 15 mL conical tubes, add 5 mL pre-warmed DMEM culture medium into each tube.
5. Add 5 μL DMSO into one tube and add 5 μL ITalk (stock concentration: 100 mM, the final working concentration: 100 μM) into another tube. Vortex the tubes.
6. Discard the original BMDMs culture medium and add 5 mL DMSO/ITalk DMEM culture medium into each dish.
7. Culture the cells in a standard humidified 37°C, 5% CO2 incubator for 12 h.
8. Harvest the cells with IP lysis buffer.
  a. Discard the medium of BMDMs.
  b. Add 3 mL ice cold PBS into the dish along the dish wall to wash cells. Discard the PBS.
  c. Add 10 μL 100× protease inhibitors into each 1 mL IP lysis buffer.
  d. Add 250 μL IP lysis buffer (pre-added 100× protease inhibitors) directly into the dish.
  e. Use a 100 μL pipettor to flush the lysis buffer up and down in the dish to make sure the lysis buffer can cover the whole area of the plate.
9. Lyse the cells on ice for 20 min and collect the lysate into new 1.5 mL tubes.

**Pause point:** You can freeze the samples at −80°C or continue to the next step.

### Click chemistry in the cell lysate

**Timing: 2 days**

This step describes the detailed methods of click chemistry in the cell lysate. This step also describes the preparation of western blot samples.

10. Sonicate the lysate in ice water for 10 min (Bioruptor plus, High power, run 30 s, rest 30 s, 10 cycles) and then centrifuge the lysate (16,000 × *g*, 4°C, 10 min) to remove precipitation of cell debris and collect the supernatant phase into new tubes.

***Alternatives:*** Other sonicators also work and this step is optional if there is no sonicator.

11. Use Pierce BCA Protein Assay Kits (Thermo Scientific) to determine the protein concentration and dilute the protein concentration to 2 mg/mL by IP lysis buffer (pre-added 100× protease inhibitors).

***Note:*** It is not necessary to strictly limit the protein concentration to 2 mg/mL. 1.2 mg/mL to 2 mg/mL is also suitable.

12. Add TCEP (5 μL), TBTA (2.5 μL), biotin-azide (2.5 μL), Cu_2_SO_4_ (5 μL) in order into 250 μL protein lysate (Table1).

**Table 1:** Reagents needed for click chemistry

Reagent	Stock concentration	Working concentration
TCEP	50 mM, dissolved in ddH_2_O	1 mM
TBTA ligand	10 mM, dissolved in DMSO	100 μM
Biotin-azide	10 mM, dissolved in DMSO	100 μM
Cu_2_SO_4_	50 mM, dissolved in ddH_2_O	1 mM

***Note:*** We have also tried to mix the TCEP, TBTA, biotin-azide and Cu_2_SO_4_ together before adding into the protein lysate. It also works.

13. Incubate samples in Eppendorf ThermoMixer C (600 rpm, 27°C) for 1 h, vortexing every 15 min.

***Alternatives:*** Incubate for 1 hour at a common rotator at 18°C–27°C, vortexing every 15 min.

***Note:*** After 1 hour reaction, the solution will become a little turbid.

14. Then centrifuge the lysis (12000 × *g*, 4°C, 5 min) to precipitate the protein. Troubleshooting 1.
15. Discard the supernatant and wash protein pellets with 250 μL ice cold methanol twice. Discard the methanol.

***Note:*** It is not possible and necessary to resuspend the pellets in the methanol.

16. Add 250 μL 1.2% SDS/PBS into each sample, and sonicate the protein pellets to completely dissolve the protein pellet in SDS buffer. Troubleshooting 2.
17. Heat the samples at 95°C for 5 min.
18. Dilute each sample to a final volume of 1.5 mL with PBS.
  a. Retain 50 μL aliquot as post-clicked input.
  b. Add 10 μL 6× protein loading buffer into each input.
  c. Heat the input at 95°C for 10 min.
  d. Store the input samples at −20°C.
19. Incubate the remainder with 10 μL streptavidin agarose beads on a rotator for about 8 h at 4°C.
20. Rotate the samples at 18°C–27°C for 2 h to resolubilize the SDS.
21. Wash beads five times with 0.2% SDS/PBS.
  a. Pellet agarose beads by centrifugation at 4000 × *g* for 3 min.
  b. Discard the supernatant.
  c. Add 1.5 mL 0.2% SDS/PBS.
  d. Invert the tube on a rotator to wash the agarose beads at 18°C–27°C for 10 min.
  e. Repeat washing for 5 times. Be careful not to aspirate the beads.
22. Wash beads a further three times with 1.5 mL ddH_2_O.

***Note:*** In the last wash, discard the wash water as dry as possible carefully. Water can be carefully removed by pipette with 20 μL tip to remove as much water as possible.

**Figure 1:**
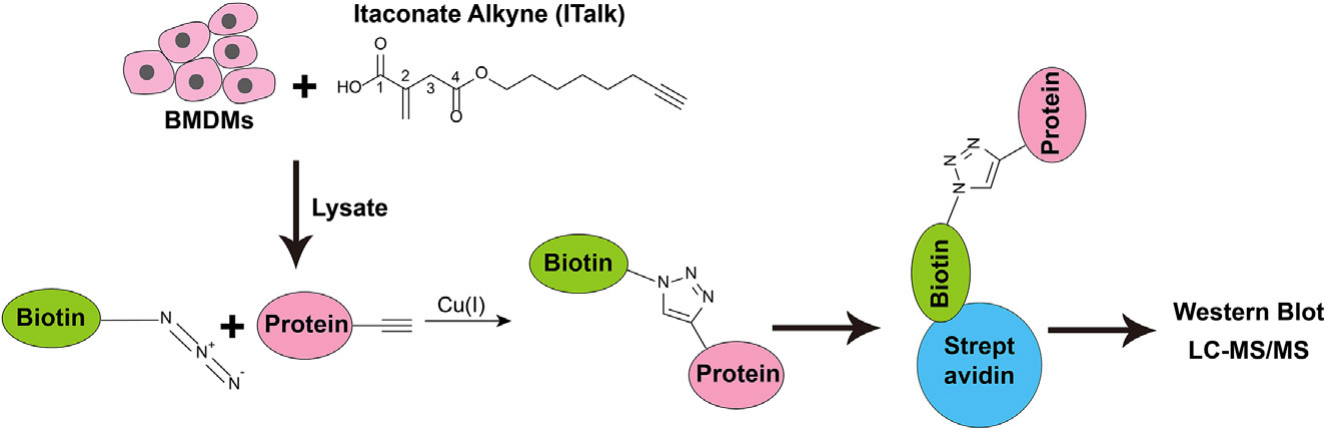
Schematic of click chemistry Primary WT BMDMs were treated with ITalk for 12 h. Lysates were subsequently Cu^+^ clicked, using a biotin-azide and streptavidin beads for pull-down of ITalk interacting proteins. Streptavidin beads were washed and eluted for immunoblot analysis or LC-MS/MS analysis.

23. Add 50 μL 1× protein loading buffer into each tube and heat the samples at 95°C for 10 min.
24. Centrifugate the samples at 12,000 × *g* for 1 min. Process for SDS-PAGE and western blot assay (Figure 1).

**Pause point:** You can freeze the samples at −20°C or continue to the next step.

***Note:*** Mass-spectrometry would also be an option to detection the 4-OI alkylated proteins. We can directly send the beads at step 22 to a mass-spectrometry lab to do in beads trypsin digestion or send the SDS-PAGE gel at step 24 to a mass-spectrometry lab to do in gel trypsin digestion to analyze the 4-OI alkylated proteins. Detailed methods can be found in these protocols or articles.^4^^,^^9^^,^^12^^,^^13^

### Detecting alkylated-proteins by western blot

**Timing: 2 days**

This step describes the detailed methods of detecting alkylated-proteins by western blot.

25. Cast a 10% (v/v) acrylamide separating gel.
  a. Mix 4.0 mL ddH_2_O, 3.3 mL 30% acrylamide, 2.5 mL 1.5 M Tris (pH = 8.8), and 0.1 mL 10% SDS in a 15 mL conical tube.
  b. Add 0.1 mL 10% APS and 6 μL TEMED.
  c. Cast about 7 mL gel within an 8.2 cm × 10.1 cm × 1.5 mm gel cassette.
  d. Gently overlay with 1 mL isopropanol to create a uniform interface above the separating gel.
26. Cast a stacking gel on top of the separating gel.
  a. After the gel has polymerized (approximately 30 min), pour off the isopropanol, wipe the remaining isopropanol with absorbent paper.
  b. For 10 mL 5% stacking gel, mix 6.8 mL ddH_2_O, 1.7 mL 30% acrylamide, 1.25 mL 1 M Tris (pH = 6.8), and 0.08 mL 10% SDS in a 15 mL conical tube.
  c. Add 0.1 mL 10% APS and 10 μL TEMED.
  d. Immediately cast about 2.5 mL stacking gel on top of the separating gel.
  e. Immediately insert the 15-well-gel comb into the stacking gel. Be careful to avoid introducing air bubbles.
27. Once the stacking gel has polymerized (approximately 30 min), place the gel into the electrophoresis unit, add 1× running buffer to gel box, fill up the tank and carefully remove the comb.
28. Load protein samples into the wells using 20 μL tips. Usually, load 20 μL sample into each well. Load 5 μL protein ladder beside the sample wells.

***Note:*** The post-click-input samples and streptavidin pull-down samples should be loaded in different gels or loaded protein ladders between them. According to our estimation, the protein mass of input samples we loaded is about 3–5 μg/well and the protein mass of streptavidin pull-down samples we loaded may be about 1–2 μg/well.

29. Close the electrophoresis unit and connect to the power supply. Electrophorese at 100 V, 1.5 h when the dye of the bromophenol blue has reached the bottom of the gel.

***Note:*** Step 25–29 is a traditional protocol of SDS-Polyacrylamide Gel Electrophoresis.

30. Traditional wet tank transfer.
  a. Cut the PVDF membrane and six pieces of thick blot filter paper to the size of the gel. Soak the PVDF membrane in 100% methanol for 1 min.
  b. Briefly submerge filter paper in 1× Transfer buffer.
  c. Following electrophoresis, carefully pry the two gel plates apart and make sure that the gel remains intact on one plate.
  d. Discard the stacking gel and transfer the remaining resolving gel to the filter paper.
  e. Carefully place two foam pads, three filter papers, resolving gel, PVDF membrane, three additional filter papers and two additional foam pads together in the transfer cassette to assemble the transfer sandwich.
    **CRITICAL:** Avoid air bubbles between each layer in this step. If necessary, remove air bubbles by rolling a wet test tube over each layer after placing it down.
  f. Place the cassette in the transfer tank and place an ice block in the tank.
  g. Add 1× transfer buffer into the tank.
  h. Close the transfer tank and connect to power supply. Transfer at 100 V, 1.5 h in a cold room or in an ice-water bath.
31. Immunoblotting. Troubleshooting 3, 4, and 5.
  a. Incubate the membrane in blocking buffer on an orbital shaker at 18°C–27°C for 1 h.
  b. Remove the blocking buffer. Dilute primary antibody in fresh blocking buffer.
    ***Note:*** Dilute SA-HRP with 1:1,500, mouse anti-β-actin primary antibody with 1:3,000 and rabbit anti-STING primary antibody with 1:1,500.
  c. Incubate membrane in primary antibody for about 8 h in 4°C on an orbital shaker.
  d. Remove the primary antibody and wash the membrane with TBST three times, for 5 min each, on an orbital shaker at 18°C–27°C.
    ***Note:*** Diluted primary antibody can be re-used for about 3-5 times, and should be stored in −20°C.
  e. Incubate membrane in the secondary antibody for 1 h at 18°C–27°C on an orbital shaker.
    ***Note:*** Dilute goat anti-mouse IgG secondary antibody in blocking buffer with 1:3000. For membrane incubated with anti-β-actin primary antibody, incubate membrane in anti-mouse IgG secondary antibody. For membrane incubated with SA-HRP, it needn’t to be incubated with the secondary antibody. Dilute goat anti-rabbit IgG secondary antibody in blocking buffer with 1:3000. For membrane incubated with anti-STING primary antibody, incubate membrane in anti-rabbit IgG secondary antibody.
  f. Remove the secondary antibody and wash the membrane with TBST three times, for 5 min each time, on an orbital shaker at 18°C–27°C.
  g. Mix SuperSignal West Pico PLUS Substrate A and B at the ratio of 1:1.
  h. Incubate the membrane with ECL substrate for 1 min.
  i. Place the membrane in the iBright 1500 (Thermo Fisher Scientific). Choose the proper exposure time to detect signals.

## Expected outcomes

**Figure 2:**
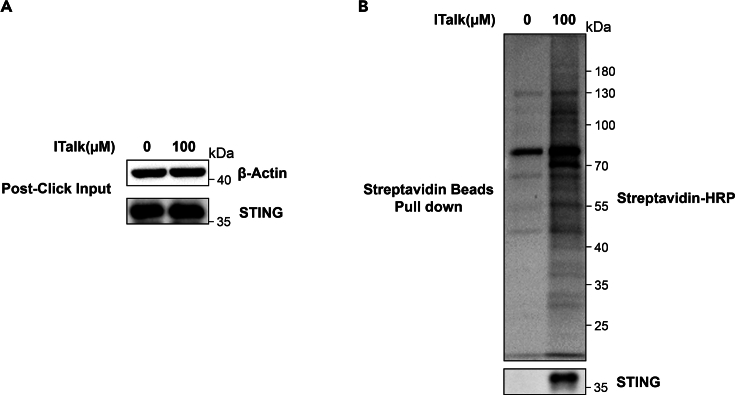
Detection of 4-octyl-itaconate-alkylated proteins in primary mouse macrophages by western blotting (A) Immunoblot analysis of post-click input. Actin is used as internal control so that the total protein amount used for click is equal between different treatments. (B) Immunoblot analysis of proteins in streptavidin pull-down from clicked lysates of BMDMs treated with ITalk or DMSO for 12 h. Streptavidin-HRP is used to probe the labeling protein. STING is an example of the probe-modified protein.

In immunoblotting experiments, we can use different primary antibodies to investigate whether the proteins we are interested in would be pulled-down by streptavidin beads (Figure 2). We can see that STING can be pulled-down by streptavidin beads. Therefore, STING can be modified by 4-octyl itaconate (Figure 2). It is also a good way to detect the pulled-down proteins by LC-MS/MS. We include the ITalk-targeted protein ID list in the Table S1. Compared with the DMSO control, the ITalk group pulled-down more proteins. Some of them are reported 4-octyl itaconate alkylated proteins, including Keap1 and so on.

## Limitations

ITalk may be hydrolyzed by esterase in some cells, which would decrease the pulling-down efficiency of streptavidin. Replacing the ester linkage of ITalk with an amide would enhance labeling efficiency in some cells.^14^ In the immunoblotting experiment, we used DMSO as a control. A saturated non-reactive click probe would serve a better control.

## Troubleshooting

### Problem 1

No protein pellets after click (step 14).

### Potential solution

Increase the protein amount used for click. Extend the centrifugation time. Add cold acetone to precipitate the proteins.

### Problem 2

Protein pellets are hard to redissolve (step 16).

### Potential solution

Pipet the pellets up and down several times to resuspend the pellet in 1.2% SDS/PBS, then sonicate pellets in SDS for another or more cycles.

### Problem 3

No or low signal from clicked samples after WB (step 31).

### Potential solution

It is possible the presence of interfering chemicals EDTA. Therefore, we should use the protease inhibitor cocktail with EDTA free to avoid the presence of EDTA in the lysis buffer.

### Problem 4

High background signal (step 31).

### Potential solution

Increase the blocking time to 2–3 h or blocking the membrane in the cold room for overnight. Increase the protein concentration used for click. Decrease the dilution rate of the primary antibody. This part may also be caused by blocking the membrane with 5% milk. Milk may naturally contain biotinylated proteins that will be detected by streptavidin-HRP antibody and lead to high background.

Blocking with 1%–2% BSA-TBST can be used as alternative if milk containing blocking buffer leads to high background.

### Problem 5

High signal in the DMSO control group (step 31).

### Potential solution

Increase the washing times in step 21. Increase the ITalk concentration used in step 5.

## Resource availability

### Lead contact

Further information and requests for resources and reagents should be directed to and will be fulfilled by the lead contact, Hang Yin (yin_hang@tsinghua.edu.cn).

### Technical contact

Technical questions on executing this protocol should be directed to and will be answered by the technical contact, Chaofei Su (scf19@mails.tsinghua.edu.cn).

### Materials availability

This study did not generate new unique reagents; materials listed in the key resources table are available for use upon request to the lead contact.

### Data and code availability

This study does not report original code. Any additional information required to reanalyze the data reported in this paper is available from the lead contact upon request.

## Acknowledgments

We thank all members in our lab for helpful discussions. This work was supported by the 10.13039/100014717National Natural Science Foundation of China (grant no. 22137004 and 82430109), the 10.13039/501100004826Natural Science Foundation of Beijing, China (grant no. IS23107) and the Beijing Outstanding Young Scientist Program, China (grant no. BJJWZYJH01201910003013).

## Author contributions

H.Y. conceptualized the project, supervised all the experiments, and reviewed the manuscript. C.S. designed and performed the experiments, interpreted the data, and wrote the manuscript. T.C. and H.Z. performed the experiments and revised the manuscript.

## Declaration of interests

The authors declare no competing interests.

## Supplemental information

Table S1. ITalk-targeted protein ID list, related to expected outcomes
